# Declining Trends of Pneumococcal Meningitis in Gambian Children After the Introduction of Pneumococcal Conjugate Vaccines

**DOI:** 10.1093/cid/ciz505

**Published:** 2019-09-05

**Authors:** Bakary Sanneh, Catherine Okoi, Mary Grey-Johnson, Haddy Bah-Camara, Baba Kunta Fofana, Ignatius Baldeh, Alhagie Papa Sey, Mahamadou Labbo Bah, Mamadi Cham, Amadou Samateh, Effua Usuf, Peter Sylvanus Ndow, Madikay Senghore, Archibald Worwui, Jason M Mwenda, Brenda Kwambana-Adams, Martin Antonio

**Affiliations:** 1 National Public Health Laboratories, Ministry of Health and Social Welfare, Kotu; 2 World Health Organization (WHO) Collaborating Center for New Vaccines Surveillance, Medical Research Council Unit The Gambia at London School of Hygiene and Tropical Medicine, Fajara, Banjul; 3 Faculty of Infectious and Tropical Diseases, London School of Hygiene and Tropical Medicine, United Kingdom; 4 Edward Francis Small Teaching Hospital, Ministry of Health and Social Welfare, Banjul; 5 WHO Country Office The Gambia, Kotu; 6 Department of Health Services, Ministry of Health and Social Welfare, Banjul, The Gambia; 7 Immunization, Vaccines and Development, WHO Regional Office for Africa, Brazzaville, Republic of Congo; 8 Microbiology and Infection Unit, Warwick Medical School, University of Warwick, Coventry, United Kingdom

**Keywords:** pediatric bacterial meningitis, *Streptococcus pneumoniae*, *Haemophilus influenzae*, *Neisseria meningitidis*, pneumococcal conjugate vaccines

## Abstract

**Background:**

Acute bacterial meningitis remains a major cause of childhood mortality in sub-Saharan Africa. We document findings from hospital-based sentinel surveillance of bacterial meningitis among children <5 years of age in The Gambia, from 2010 to 2016.

**Methods:**

Cerebrospinal fluid (CSF) was collected from children admitted to the Edward Francis Small Teaching Hospital with suspected meningitis. Identification of *Streptococcus pneumoniae* (pneumococcus), *Neisseria meningitidis* (meningococcus), and *Haemophilus influenzae* was performed by microbiological culture and/or polymerase chain reaction where possible. Whole genome sequencing was performed on pneumococcal isolates.

**Results:**

A total of 438 children were admitted with suspected meningitis during the surveillance period. The median age of the patients was 13 (interquartile range, 3–30) months. Bacterial meningitis was confirmed in 21.4% (69/323) of all CSF samples analyzed. Pneumococcus, meningococcus, and* H. influenzae* accounted for 52.2%, 31.9%, and 16.0% of confirmed cases, respectively. There was a significant reduction of pneumococcal conjugate vaccine (PCV) serotypes, from 44.4% in 2011 to 0.0% in 2014, 5 years after PCV implementation. The majority of serotyped meningococcus and* H. influenzae* belonged to meningococcus serogroup W (45.5%) and* H. influenzae* type b (54.5%), respectively. Meningitis pathogens were more frequently isolated during the dry dusty season of the year. Reduced susceptibility to tetracycline, trimethoprim-sulfamethoxazole, and chloramphenicol was observed. No resistance to penicillin was found.

**Conclusions:**

The proportion of meningitis cases due to pneumococcus declined in the post-PCV era. However, the persistence of vaccine-preventable meningitis in children aged <5 years is a major concern and demonstrates the need for sustained high-quality surveillance.

## Teaser

The proportion of meningitis cases due to pneumococcus in children has declined post–pneumococcal conjugate vaccine introduction in The Gambia. However, the persistence of vaccine-preventable bacterial meningitis in children aged <5 years is a major concern and warrants further investigation.

Bacterial meningitis is associated with very high fatality, with reported annual mortality incidence in developing countries of 98 per 100 000 in children aged <1 year [1, 2]. Outbreaks of epidemic meningitis are particularly frequent in the African meningitis belt, a region that extends from Senegal in the west, to Ethiopia and Eritrea in the east [3–6]. Predominant bacterial causes of meningitis include *Neisseria meningitidis* (meningococcus), *Streptococcus pneumoniae* (pneumococcus), and *Haemophilus influenzae* type b (Hib) in the pre–Hib vaccine era [1, 5, 7, 8].

Immunization against pneumococcus, meningococcus, and Hib is one of the proven ways of reducing morbidity and mortality of childhood bacterial meningitis [8, 9]. To this end, initiatives such as Gavi, the Vaccine Alliance and the Meningitis Vaccine Project have led to the accelerated introduction of pneumococcal, Hib, and meningococcal conjugate vaccines in several African countries during the last 10–15 years. In The Gambia, Hib vaccine was introduced into the routine immunization schedule for children in 1997 as a primary 3-dose course at 2, 3, and 4 months with no booster. Likewise, conjugate vaccine against pneumococcus was introduced in 2009 without a catch-up in a 3 + 0 schedule, with doses given at 2, 3, and 4 months. Coverage rates for pneumococcal and Hib conjugate vaccines remained high (>95%) throughout the surveillance period. Meningococcus vaccination campaigns targeting meningococcus serogroup A were conducted in 2013 for individuals aged 1–29 years. However, monitoring impact of new vaccine efficacy is reliant on robust and high-quality surveillance data, which are lacking in many low-income countries including The Gambia.

We have conducted analysis to describe the epidemiology and etiology of suspected meningitis among children aged <5 years in The Gambia between 2010 and 2016.

## METHODS

### Study Area and Design

Hospital-based sentinel surveillance of bacterial meningitis among children <5 years of age is ongoing at the Edward Francis Small Teaching Hospital (EFSTH) situated in Banjul, the capital city of The Gambia. EFSTH is the only tertiary care and referral facility in the country and receives patients from all health facilities nationwide. The population of The Gambia is estimated as 1.859 million [10]. The prevalence of human immunodeficiency virus has remained relatively low, at 1.8% (95% CI: 1.4%–3.3%), with predominance in urban rather than rural populations [11]. The climate is subtropical with distinct dry and rainy seasons. The *harmattan* (hot and dry winds) lasts from November until the onset of the rains in mid-May.

### Patients

All suspected cases of meningitis admitted at the EFSTH pediatric ward between 2010 and 2016 underwent a lumbar puncture as part of routine diagnostic procedures. A suspected meningitis case was defined as sudden onset of fever (>38°C axillary or >38.5°C rectal temperature), with a combination of any of the following clinical symptoms: altered consciousness, stiff neck, sensitivity to light, and bulging of the fontanelle if the child is <1 year old; a confirmed case was defined as any suspected meningitis case that is laboratory-confirmed by culturing or identifying (ie, by polymerase chain reaction [PCR]) a bacterial pathogen (*H. influenzae*, pneumococcus, or meningococcus) in cerebrospinal fluid (CSF) or blood in a child with a clinical syndrome consistent with bacterial meningitis [12].

### Ethical Review

Pediatric bacterial meningitis surveillance is part of routine surveillance for invasive bacterial vaccine-preventable diseases. The standard operating procedures and protocols were approved by the Joint Ethics Committee of The Gambia government/Medical Research Council Unit The Gambia at the London School of Hygiene and Tropical Medicine (MRCG). 

Ethical approval was not a requirement in The Gambia for routine meningitis surveillance, including drug susceptibility testing of collected isolates, as this is approved within the routine diagnostic algorithm at the Ministry of Health. However, informed consent was sought from the parents or guardians of the surveillance participants. Additionally, the surveillance received overarching ethical approval (SCC1188) by the joint Medical Research Council/The Gambia government ethics board that allowed the analysis of collected West African isolates at MRCG.

### Bacteriologic Analysis of CSF Samples

Gram stain and rapid diagnostic tests for presumptive identification of pneumococcus, meningococcus, and *H. influenzae* were performed at the microbiology laboratories of the EFSTH sentinel site. In brief, a portion of the CSF specimen was centrifuged at 10 000 rpm for 10 minutes. Smears were prepared from the sediment for Gram stain following the WHO protocol [13]. Latex agglutination was performed from the CSF supernatant using Pastorex meningitis kit (Bio-Rad) for detecting Hib, pneumococcus, and meningococcus groups A, B, C, W, and Y antigens following the manufacturer’s instructions. Additionally, 0.5 mL of the uncentrifuged CSF was used to test for the presence of pneumococcus antigen using the BINAX NOW kit (Alere) when available, and white blood cell (WBC) count was carried out with the aid of an improved Neubauer counting chamber. CSF protein and glucose were analyzed using the trichloroacetic acid turbid metric [14] and glucose oxidase [15] methods, respectively.

For culture, a loopful of the CSF sediment was streaked on Columbia blood agar (BA) and chocolate agar (CA), each enriched with 5% sheep blood. Following overnight incubation, the CA and BA plates were examined for characteristic growth of pneumococcus, meningococcus, *H. influenzae*, and other pathogens. Suspected pneumococcal isolates were confirmed by optochin test (5 μg optochin disk; Oxoid). All suspected *H. influenzae* and meningococcal isolates were subjected to biochemical confirmation using analytical profile index (API NH, bioMérieux). Confirmed pneumococcal isolates were serotyped by the latex agglutination technique as described elsewhere [16]. Meningococcus serogroups were confirmed by slide latex agglutination serogrouping using Pastorex (Bio-Rad). *Haemophilus influenzae* serotyping was performed by slide agglutination using antisera for serotypes a through f (Murex Biotech). Antibiotic susceptibility tests were performed by the disk and Etest diffusion methods for commonly prescribed antibiotics in the subregion, according to Clinical and Laboratory Standards Institute guidelines [17]. All confirmed isolates were stored in 16% glycerol broths for further testing at the WHO regional reference laboratory (RRL).

### Real-time PCR Detection and Serotyping of Pathogens

Up to 1000 μL of each CSF specimen was transported to the WHO RRL for confirmatory testing by real-time PCR. Species-specific PCR assays for detection of pneumococcus, meningococcus, and *H. influenzae* were conducted as described elsewhere [5]. RNAse P gene assay was performed on all CSF specimens to confirm that samples were of human origin and to monitor the efficiency of amplification. The amplification cycle involved an initial denaturation step at 95°C for 10 minutes, followed by 45 cycles of 95°C for 15 seconds and 60°C for 1 minute. Positivity for each of the targets was deduced using cycle threshold (Ct) values, and Ct values of ≤36 were considered positive.

### Serogroup- and Serotype-specific Quantitative PCR Assays

Meningococcal serogrouping and *H. influenzae* serotyping were performed as previously described [18]. Targets for the mentioned pathogens included *sacB*, *synD*, *synE*, *synG*, *xcbB*, and *synF* genes for serogroups A, B, C, W, X, and Y, respectively. For *H. influenzae*, the following genes were screened for serotyping: *acsB* (type a), *bcsB* (Hib), *ccsD* (type c), *dscE* (type d), *ecsH* (type e), and *bexD* (type f). Ct values of ≤36 were considered positive.

### *Streptococcus pneumoniae* Serotyping

In preparation for nucleic acid extraction for pneumococcal serotyping, 200 µL of CSF was added to 50 µL of TE buffer containing 0.08 g/mL of lysozyme (Sigma, L-6876) and 150 U/mL of mutanolysin (Sigma, M-9901), and the mixture was incubated for 1 hour at 37°C. The remaining extraction procedures followed the Qiagen DNA Mini kit manufacturer’s instructions. Purified DNA extracts were subjected to sequential triplex quantitative PCR (qPCR) assay for detecting 21 pneumococcal capsular serotypes for the African scheme as previously described [19]. Nontypeable pneumococci with Ct values ≤32 by qPCR were further subjected to conventional multiplex serotyping PCR assays.

### Whole Genome Sequencing

Whole genome sequencing (WGS) was performed at the Sanger institute on an Illumina Hiseq as previously described [20]. Sequencing reads were mapped on the pneumococcus American Type Culture Collection (ATCC) 700669 reference genome. The phylogeny of the tree was reconstructed from variable sites using RAxML [21] and visualized in iTOL [22].

## RESULTS

### Patient Population and Characteristics

A total of 438 suspected meningitis cases were reported at EFSTH between 2010 and 2016. The demographic and clinical characteristics of the children with suspected meningitis are shown in Table 1. The median age of the patients was 13 (interquartile range, 3–30) months. Almost two-thirds of the patients were male and two-fifths were aged <1 year with a mortality rate of 13.9% (61/438). More than half of the patients had CSF with clear appearance as well as with WBC counts (67.4%) of ≤10 cells/μL. Nearly all (394/438) of the cases did not have a known clinical diagnosis reported.

**Table 1.** Patient Characteristics of Suspected Bacterial Meningitis Cases (N = 438)

Characteristic	No. (%)
Age, mo	
0–11	178 (40.6)
12–23	82 (18.7)
24–59	131 (29.9)
Unknown	47 (10.7)
Sex	
Male	260 (59.4)
Female	174 (39.7)
Unknown	4 (0.9)
Antibiotic use before admission	
Yes	19 (4.3)
No	71 (16.2)
Unknown	348 (79.5)
Outcome	
Discharged alive	356 (81.3)
Died	61 (13.9)
Unknown	21 (4.8)
CSF appearance	
Clear	242 (55.3)
Turbidity	58 (13.4)
Xanthochromic	20 (4.6)
Bloodstained	51 (11.6)
Unknown	67 (15.3)
WBC count, cells/μL	
0	3 (0.68)
≤10	295 (67.4)
>10 to 100	40 (9.1)
>100	22 (5.0)
Unknown/not done	78 (17.8)
Case type	
Suspected meningitis	369 (84.2)
Confirmed meningitis	69 (15.8)

Abbreviations: CSF, cerebrospinal fluid; WBC, white blood cell.

### Bacterial Identity and Serotypes

**Figure 1.**
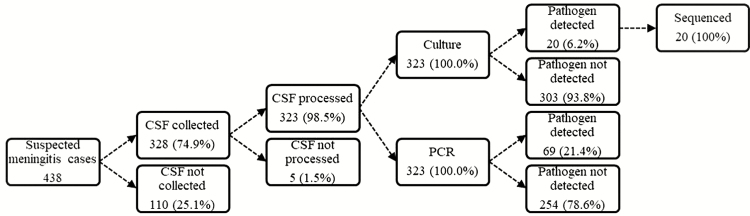
Summary of cerebrospinal fluid collection and processing for invasive bacterial disease surveillance in The Gambia (2010–2016). Abbreviations: CSF, cerebrospinal fluid; PCR, polymerase chain reaction.

**Figure 2.**
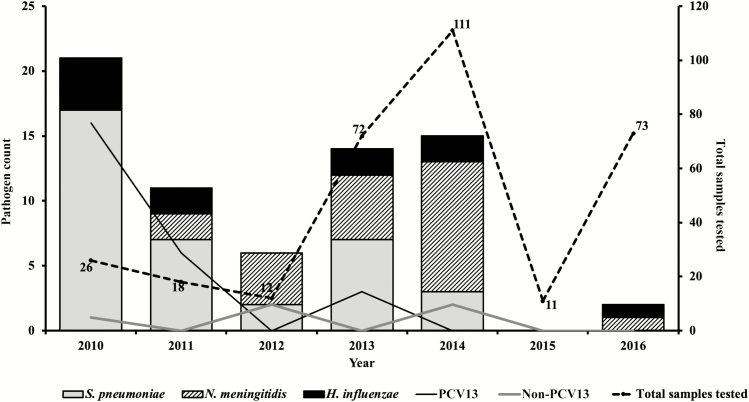
Distribution of pathogens associated with pediatric bacterial meningitis in The Gambia (2010–2016). Thirteen-valent pneumococcal conjugate vaccine (PCV13) types include serotypes 1, 5, 6A/6B, 14, 19A, and 23F; non-PCV13 serotypes include 8, 11A, and 3 nontypeable pneumococci by real-time polymerase chain reaction.

Overall, 98.5% (323/328) of CSF specimens were tested by rapid antigen tests, culture, and PCR assays (Figure 1). A fifth of the CSF specimens tested (69/323) had laboratory-confirmed bacterial meningitis. Pneumococcus was the major pathogen, accounting for 52.2% (36/69) of all confirmed cases, followed by meningococcus (31.9% [22/69]) and *H. influenzae* (16.0% [11/69]) (Figure 2). Prior to 2014, pneumococcal serotypes 1, 5, and 6A/6B were the most common pathogens among confirmed cases. No cases of pneumococcal meningitis were found during 2015 and 2016, the last 2 years of the surveillance (Figure 2).

**Figure 3.**
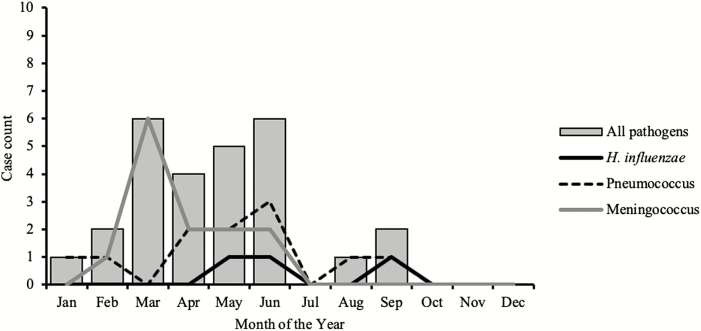
Seasonality of meningitis pathogens in The Gambia (2010–2016).

Almost half (10/22) of meningococcal cases belonged to meningococcus serogroup W, and Hib accounted for 54.5% (6/11) of all *H. influenzae* serotypes. Meningitis pathogens were more frequently detected during the dry season of January to June compared to the wet season (Figure 3).

### Genotypes and Resistance Patterns

**Figure 4.**
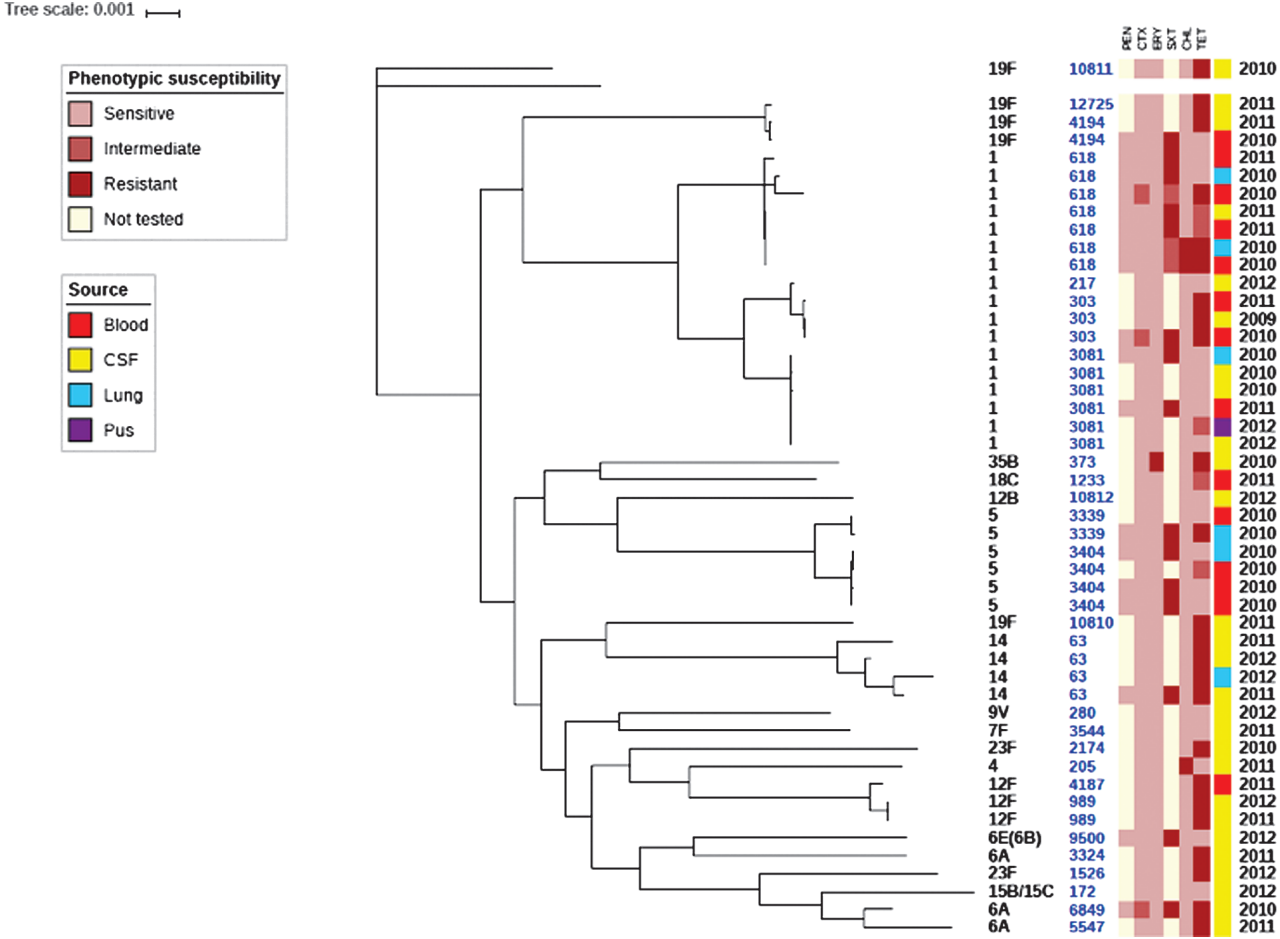
Phylogeny and antibiogram of pneumococcal isolates associated with childhood bacterial meningitis. Isolates cultured from blood (n = 14); lungs (n = 6), and pus (n = 1) are included to contextualize the isolates cultured from cerebrospinal fluid specimens (n = 20). Resistance and intermediate resistance were confirmed by Etest. Abbreviations: CHL, chloramphenicol; CSF, cerebrospinal fluid; CTX, cefotaxime; ERY, erythromycin; PEN, penicillin; SXT, trimethoprim-sulfamethoxazole; TET, tetracycline.

The EFSTH sentinel site sent a total of 41 pneumococcal isolates to the WHO RRL for confirmation, serotyping, antibiogram, and molecular characterization during the surveillance period. These isolates were primarily from CSF specimens from suspected cases of meningitis but also included isolates from blood, lung aspirate, and pus for reference. WGS analysis of the pneumococcal isolates revealed 3 major clades (Figure 4): clade 1, 19F; clade 2, 19F and serotype 1; clade 3, all other serotypes including some 19F. Additionally, isolates belonging to the same serotype clustered together, particularly the predominant invasive serotypes in The Gambia (serotypes 1, 5, and 14). However, serotype 19F appeared in all the 3 clades, especially ST10810, which clustered with serotype 14 ST63. High susceptibility to penicillin, cefotaxime, and erythromycin was noted. The only erythromycin-resistant isolate (minimum inhibitory concentration = 125 µg/mL) was a serotype 35B (nonvaccine serotype). Reduced susceptibility was noted to tetracycline, co-trimoxazole, and to a lesser extent, chloramphenicol. Compared to the pneumococcal serotype 1 ST3081 isolates, ST618 isolates were more resistant to trimethoprim-sulfamethoxazole (16% [1/6] vs 57% [4/7], respectively).

## DISCUSSION

We report hospital-based sentinel surveillance data on the etiology and trends of vaccine-preventable bacterial meningitis among children <5 years old in The Gambia from 2010 through 2016. Pneumococcus was the predominant etiologic agent in confirmed meningitis cases. Pneumococcal vaccine serotypes decreased, whereas the proportion of nonvaccine types increased during the surveillance period. Hib remains a cause of bacterial meningitis among young children despite the widespread use of Hib conjugate vaccine.

The widespread use of PCVs has been shown to markedly reduce carriage of pneumococcal vaccine serotypes among both vaccinated and unvaccinated individuals [23–26]. The 13-valent pneumococcal conjugate vaccine (PCV13) was introduced into The Gambia’s Expanded Programme on Immunization in 2011. Recently, a population-based study of invasive pneumococcal disease conducted in the Upper River Region of The Gambia showed remarkable public health successes of PCVs associated with a reduction in invasive pneumococcal diseases [9]. Although the number of samples is small, we showed a marked reduction in the number of vaccine serotypes and an increase in the proportion of nonvaccine serotypes prior 2014. Additionally, there was no case of pneumococcal meningitis during the last 2 years (2015 and 2016) of the surveillance period. The majority of CSF samples were collected during the post-PCV13 era.

Interestingly, our surveillance data showed that *H. influenzae* meningitis was not seasonal, unlike pneumococcus, which peaked in the dry season. In contrast, meningococcal meningitis was seasonal and appeared to mimic pneumococcal disease. Meningococcus serogroup W caused several sporadic cases of meningitis in The Gambia in the 1990s [27] and was also isolated during a serogroup A outbreak in Mali in 1994 [28]. Although numbers are small, the predominance of serogroup W is consistent with previous reports and suggests continued low-level incidence of serogroup W in The Gambia. This finding requires a strategy for enhanced surveillance, epidemic detection and control, and a revision of the vaccination policy in The Gambia. Likewise, Hib was among the leading causes of bacterial meningitis in children aged <5 years prior to the introduction of the Hib conjugate vaccine in The Gambia [29]. Studies of the efficacy of the vaccine in The Gambia’s Western Region showed that it was 95% effective in preventing meningitis and bloodstream infection, and 100% effective in preventing pneumonia [8, 30]. It is therefore concerning that Hib still causes a substantial proportion of bacterial meningitis in The Gambia, with 6 cases detected throughout the surveillance period.

Finally, our finding of seasonality in meningitis causing pathogens agrees with the pattern seen in the meningitis belt, with peaks in the first 5 months of the year [5, 31, 32]. A study in The Gambia pre-PCV showed regular epidemics of respiratory syncytial virus outbreaks mainly during the rainy season interrupted by periods of irregularity. These epidemics coincided with an increase in admissions due to respiratory tract infections [33]. Meningitis outbreaks occur during the dry and dusty harmattan winds that blow between November and May across the Sahel. These harmattan winds carry fine fractions of particulate matter. Following the introduction of conjugate vaccines, the links between dust inhalation, carriage of respiratory pathogens including influenza, and respiratory syncytial virus need to be investigated further in The Gambia.

A major limitation of the surveillance is the incomplete clinical and demographic data for up to 40% of patients with suspected meningitis who had a lumbar puncture performed. This reduced the quality of data available to describe the basic clinical characteristics and etiology of pediatric meningitis cases. Additionally, incomplete vaccination records, especially for *H. influenzae* meningitis cases, hampered our description of vaccination failure in these children. Also, it would have been very useful to know what percentage of the patients with pneumococcal meningitis had received PCV vaccine. Unfortunately, these data were not available for such an analysis.

## CONCLUSIONS

Meningitis caused by pneumococcus, meningococcus, and *H. influenzae* remains a major cause of child mortality and morbidity in The Gambia. Enhanced surveillance for monitoring of vaccine impact and detection of meningitis is recommended for early detection of epidemics. The WHO RRL should continue to work with The Gambia sentinel site to strengthen laboratory and surveillance capacity that will enhance high-quality data capture on bacterial meningitis–causing pathogens.

## Notes

***Invasive Bacterial Disease (IBD) Writing Group*** ***members.*** Brenda Kwambana-Adams, Madikay Senghore, Effua Usuf, Archibald Worwui, Egere Uzochukwu, Akram Zaman, Catherine Okoi, Florian Gehre, Leopold Tientcheu, Nuredin Ibrahim Mohammed, Felix Dube, Peter Ndow, Sambou Suso, Sheikh Jarju, Dam Khan, Ebruke Chinelo, Rowan Bancroft, Jason M. Mwenda, and Martin Antonio.

***Author contributions.*** M. A. and J. M. M. established the WHO/AFRO-supported Paediatric Bacterial Meningitis Surveillance Network in West Africa. M. A. supervised the overall network, including setting up the sentinel surveillance system. B. S., M. G. J., H. F., B. F., I. B., A. P. S., M. L. B., M. C., and A. S. clinically investigated and recruited the patients at the sentinel sites, collected demographic data, performed microbiological testing at sentinel sites, and shipped cerebrospinal fluid and bacterial isolates to the WHO RRL at MRCG for confirmatory testing and molecular analysis, supervised by B. K. A. and M. A. In addition, B. K. A. and M. A. developed the analysis plan and contributed to analysis and interpretation of data along with the IBD Writing Group. C. O., B. K. A., and M. A. drafted the manuscript. All authors contributed to the interpretation of the findings and the writing of the final manuscript.

***Acknowledgments.*** The authors thank Kebba Gibba of World Health Organization Country Office. The Gambia for his immense support during implementation of the pediatric bacterial meningitis (PBM) surveillance; the WHO Intercountry Support Team for coordination, advice, and support throughout the project; the surveillance participants and their families in The Gambia; and the staff members and students at the WHO regional reference laboratory (RRL) at the Medical Research Council The Gambia (MRCG) as well as the IBD writing group for their advice and input. The authors also thank Ebrima Barrow, Omar Jallow, Dr Mariama Sonko, and the rest of the clinical and laboratory staff at Edward Francis Small Teaching Hospital, who contributed to the success of this PBM surveillance.

***Financial support.*** Financial support for sentinel site surveillance was provided by The Gambia Ministry of Health, Gavi - the Vaccine Alliance, through a grant to the WHO for the African Paediatric Bacterial Meningitis Surveillance Network.

***Supplement sponsorship.*** This supplement was supported with funds from Gavi, the Vaccine Alliance through The World Health Organization and the CDC Foundation, and The Medical Research Council Unit The Gambia at the London School of Hygiene and Tropical Medicine.

***Potential conflicts of interest.*** E. U. has received fees from a GlaxoSmithKline consultancy (2014–2017). All other authors report no potential conflicts of interest. All authors have submitted the ICMJE Form for Disclosure of Potential Conflicts of Interest. Conflicts that the editors consider relevant to the content of the manuscript have been disclosed.
